# Efficacy of IL-23 inhibitors in axial involvements of chronic non-bacterial osteitis with palmoplantar pustulosis: a case series

**DOI:** 10.1093/rheumatology/keaf088

**Published:** 2025-02-11

**Authors:** Hirotaka Yamamoto, Yoshinori Taniguchi, Shigeyoshi Tsuji, Philip S Helliwell, Mitsumasa Kishimoto

**Affiliations:** Department of Endocrinology, Metabolism, Nephrology and Rheumatology, Kochi Medical School Hospital, Kochi University, Nankoku, Kochi, Japan; Department of Endocrinology, Metabolism, Nephrology and Rheumatology, Kochi Medical School Hospital, Kochi University, Nankoku, Kochi, Japan; Department of Rehabilitation, Orthopedic Surgery, Psoriasis Center, Public Interest Incorporated Foundation of the Nippon Life Saiseikai Foundation, Nippon Life Hospital, Osaka, Japan; Leeds Institute of Rheumatology and Musculoskeletal Medicine, University of Leeds, Leeds, UK; Department of Nephrology and Rheumatology, Kyorin University School of Medicine, Tokyo, Japan

Rheumatology key message

- IL-23 inhibitors are effective in treating axial involvements of chronic non-bacterial osteitis with palmoplantar pustulosis.

Dear Editor, Chronic non-bacterial osteitis (CNO) is an autoinflammatory bone disorder characterized by sterile bone inflammation, encompassing the previously named pustulotic arthro-osteitis (PAO) and SAPHO syndrome. These conditions share overlapping clinical, radiological, and pathological features, including sterile osteitis, hyperostosis, and inflammatory lesions, which often affect the axial skeleton and peripheral sites [1]. Recent studies have reported that 28.6% of patients with palmoplantar pustulosis (PPP) develop CNO (CNO/PPP) [2]. Further, ∼80% of patients with CNO/PPP have anterior thoracic wall lesions, whereas 30% have axial lesions in the spinal and pelvic regions [2]. Although the mechanism of pathogenesis is unclear, the IL-12/Th1 and IL-23/17 pathway is closely involved in the pathogenesis of CNO and PPP. In recent years, IL-12/23 or IL-23 inhibitors have shown efficacy as therapies targeting key cytokines involved in CNO/PPP [3]. In fact, only IL-23 inhibitors of biologic DMARDs are approved for the treatment of PPP in Japan. However, there have been no studies showing the efficacy of IL-23 inhibitors for axial lesions in CNO/PPP.

This case series aimed to evaluate the efficacy of IL-23 inhibitors for axial involvement in CNO/PPP. Charts were retrospectively reviewed for five patients receiving IL-23 inhibitors for the treatment of CNO/PPP with axial involvement. The AS DAS with CRP (ASDAS-CRP) and modified BASDAI (mBASDAI) were used to evaluate clinical symptoms and disease activity [4]. Moreover, MRI findings before and after treatments with IL-23 inhibitor were compared.

The clinical and demographic details of the five patients are given in Supplementary Table S1, available at *Rheumatology* online. The patients’ ages ranged from 22 to 70 years (median, 58 years), and the male:female ratio was 1:4. Two of the five patients had a history of smoking. Peripheral lesions were observed in three cases. The duration of PPP and CNO from onset to initial consultation was 4 months to 27 years (median, 3 years) and 3 months to 3 years (median, 5 months), respectively. All patients except one had received dental treatment, antibiotics, NSAIDs, conventional synthetic DMARDs (csDMARDs) or TNF inhibitors prior to the initiation of IL-23 inhibitors but showed a poor response. All cases had active spinal inflammation, but no active sacroiliitis. Of five cases, three cases were treated concurrently with NSAIDs. However, two cases were treated by single administration of IL-23 inhibitor. After initiating IL-23 inhibitors, skin lesions and back pain were ameliorated in all cases. In particular, osteitis symptoms were completely relieved in all cases within 7 months–1 year of treatment with the IL-23 inhibitor. Moreover, within 1–2 years of treatment with the IL-23 inhibitor, bone marrow oedema on spinal MRI was dramatically reduced in all cases (Fig. 1B, D, F, H, J, L) compared with the findings before treatment (Fig. 1A, C, E, G, I, K; yellow arrows). The ASDAS-CRP score before treatment was 1.8–3.5 (median: 2.12), and the post-treatment score [0.6–2.0 (median: 0.96)] showed improvement:. The pretreatment mBASDAI scores were 2.1–7.2 (median: 6.5), and the post-treatment scores [0.6–1.2 (median: 1.2)] showed improvement.

**Figure 1.**
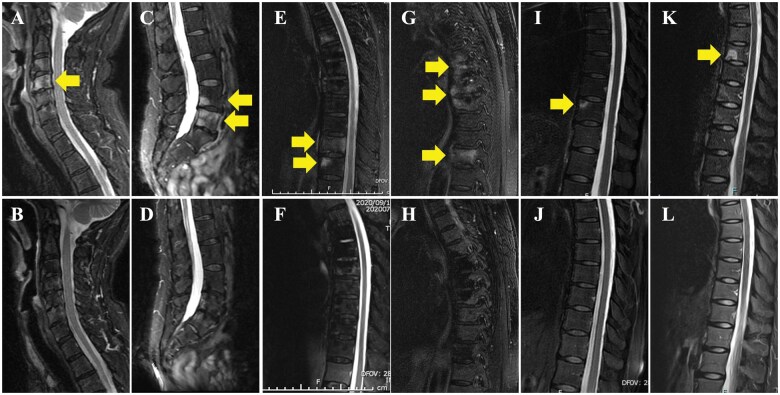
Fat-suppressed T2 imaging before and after treatment with IL-23 inhibitors. Active spondylitis was revealed before treatment with IL-23 inhibitors in cases 1 (A, cervical spine and C, lumbar spine), 2 (E, thoracic spine), 3 (G, thoracic spine), 4 (I, thoracic spine) and 5 (K, thoracic spine). Active spondylitis improved after treatment with IL-23 inhibitors in cases 1 (B and D), 2 (F), 3 (H), 4 (J) and 5 (L)

In CNO, the excessive production of IL-1β due to the activation of the NLRP3 inflammasome induces the secretion of IL-23, and the inflammatory pathway mediated by the IL-23/IL-17 axis promotes the progression of bone lesions [1]. Additionally, the IL-23/IL-17 axis plays an important role in the abnormal differentiation of keratinocytes and the maintenance of chronic skin lesions in PPP [1]. Thus, CNO and PPP share the IL-23/IL-17 axis as a pivotal target pathway, providing a basis for explaining inflammation and disease progression.

Based on these cytokine mechanisms, expert consensus recommendations for CNO therapy in adults were established in 2024 [5]. Among these recommendations, IL-23 inhibitors are included as third-line therapy and have shown potential for addressing both skin and bone symptoms, particularly in patients with PPP; however, supporting evidence remains limited for their use in treating axial involvement in CNO/PPP.

In contrast, a single case report by Yamamoto *et al.* demonstrated the potential efficacy of guselkumab in a patient with AS-type PAO (CNO/PPP) [6]. This further supports the efficacy of IL-23 inhibitors for axial involvement of CNO/PPP, as demonstrated in our case series. Why was a difference in the therapeutic response to IL-23 inhibitors observed between axial lesions in CNO/PPP and axial SpA (axSpA)? One hypothesis suggests that inflammation in CNO/PPP might mainly be driven by IL-23, whereas axSpA involves both IL-23–dependent and IL-23–independent IL-17 pathways [7]. Furthermore, important insights into axial PsA have been obtained with post-hoc analyses conducted after the PSUMMIT and DISCOVER trials [8], which demonstrated that IL-23 inhibitors improve BASDAI and ASDAS scores and normalize IL-17–related gene expression in axial PsA patients. Similar immunobiological pathways to those of axial PsA may be involved in CNO/PPP.

In conclusion, IL-23 inhibitors represent a promising therapeutic approach for the management of axial involvement in patients with CNO/PPP. Further prospective case studies are needed to confirm these findings and their efficacies.

## Supplementary Material

keaf088_Supplementary_Data

## Data Availability

The data underlying this article are available in the article and in its online supplementary material.

## References

[1] Furer V , Kishimoto M, Tomita T et al Current and future advances in practice: SAPHO syndrome and chronic non-bacterial osteitis (CNO). Rheumatol Adv Pract 2024;8:rkae114. 39411288 10.1093/rap/rkae114 PMC11474108

[2] Yamamoto T , Hiraiwa T, Tobita R et al Characteristics of Japanese patients with pustulotic arthro-osteitis associated with palmoplantar pustulosis: a multicenter study. Int J Dermatol 2020;59:441–4. 31985054 10.1111/ijd.14788

[3] Bissonnette R , Nigen S, Langley RG et al Increased expression of IL-17A and limited involvement of IL-23 in patients with palmo-plantar (PP) pustular psoriasis or PP pustulosis; results from a randomised controlled trial. J Eur Acad Dermatol Venereol 2014;28:1298–305. 24112799 10.1111/jdv.12272

[4] Yamanaka K , Okubo Y, Yasuda I et al Efficacy and safety of risankizumab in Japanese patients with generalized pustular psoriasis or erythrodermic psoriasis: primary analysis and 180-week follow-up results from the phase 3, multicenter IMMspire study. J Dermatol 2023;50:195–202. 36514850 10.1111/1346-8138.16667 PMC10107196

[5] Winter EM , Dekkers OM, Andreasen CM et al Expert consensus recommendations for the diagnosis and treatment of chronic non-bacterial osteitis (CNO) in adults. Ann Rheum Dis 2025;84:169–87. 39919892 10.1136/ard-2024-226446

[6] Yamamoto T. Effects of guselkumab on ankylosing spondylitis-type pustulotic arthro-osteitis in a patient with palmoplantar pustulosis. Dermatol Ther 2020;33:e14088. 33439526 10.1111/dth.14088

[7] Mease P , van den Bosch F. IL-23 and axial disease: do they come together? Rheumatol (Oxf Engl) 2021;60:iv28–iv33. 10.1093/rheumatology/keab617 PMC8527241 34668015

[8] Mease PJ , Helliwell PS, Gladman DD et al Efficacy of guselkumab on axial involvement in patients with active psoriatic arthritis and sacroiliitis: a post-hoc analysis of the phase 3 DISCOVER-1 and DISCOVER-2 studies. Lancet Rheumatol 2021;3:e715–23. 38287608 10.1016/S2665-9913(21)00105-3

