# Supplementary material for: Efficacy of IL-23 inhibitors in axial involvements of chronic non-bacterial osteitis with palmoplantar pustulosis: a case series
Source: Rheumatology (Oxford). 2025 Feb 11;64(6):4081–3. doi: 10.1093/rheumatology/keaf088 (PMC12107040; doi:10.1093/rheumatology/keaf088)
Supplement: keaf088_Supplementary_Data [file keaf088_supplementary_data.docx]

**Supplementary Table S1.** The characteristics of enrolled subjects treated with IL-23 inhibitors.

| Subject | 1 | 2 | 3 | 4 | 5 |
| --- | --- | --- | --- | --- | --- |
| Age/sex | 70y/F | 60y/M | 58y/F | 43/F | 22/F |
| Smoking status | Never | 40 cigarettes/day | 20 cigarettes/day | Never | Never |
| Duration of PPP/CNO | 27year/3year | 3year/5month | 1.5year/5month | 3year/2year | 4month/3month |
| Joint involvement | Sternoclavicular joint, Shoulder, Elbow, Ankle, Skull, Spine | Sternoclavicular joint, Spine | Sternoclavicular joint, Spine | Mandible, Sternoclavicular joint, Spine | Sternoclavicular joint, Shoulder, Spine |
| Failed systemic therapies | PSL, MTX, Antibiotics | NSAIDs, MTX, Antibiotics, TNF inhibitors (ADA, GLM) | NSAIDs, MTX, Sulfasalazine, Antibiotics | NSAIDs, Sulfasalazine, Bisphosphonates, TNF inhibitors (ETN, GLM) | NSAIDs, MTX, Sulfasalazine |
| ASDAS-CRP (pre) | 3.5 | 2.4 | 1.8 | 2.12 | 1.6 |
| ASDAS-CRP (post) | 2 | 0.7 | 0.6 | 0.96 | 1.1 |
| mBASDAI (pre) | 4.1 | 6.5 | 7.2 | 6.6 | 2.1 |
| mBASDAI (post) | 0.6 | 0.8 | 1.2 | 1 | 1.2 |
| Present therapy | IL-23 inhibitors | IL-23 inhibitors | IL-23 inhibitors | IL-23 inhibitors | IL-23 inhibitors |
| Adverse event | No | No | No | No | No |
| Time to relief of osteitis symptoms after initiation of IL-23 inhibitor | 1 year | 7 months | 1 year | 1 year | 8 months |
| Time to MRI improvement after initiation of IL-23 inhibitor | 1.5 years | 1 year | 1.5 years | 2 years | 1.6 years |
| Spinal MRI findings before IL-23 inhibitors (Figure number) | A, C | E | G | I | K |
| Spinal MRI findings after IL-23 inhibitors (Figure number) | B, D | F | H | J | L |
